# Absence of *Trichinella* spp. larvae in carcasses of road-killed wild animals in Paraná state, Brazil

**DOI:** 10.1590/S1984-29612022054

**Published:** 2022-10-21

**Authors:** Rafaela Maria Boson Jurkevicz, Douglas Aparecido da Silva, José Maurício Ferreira, Andressa Maria Rorato Nascimento de Matos, Bárbara Giglio Pires, Aline Ticiani Pereira Paschoal, Fernanda Pinto-Ferreira, Ana Paula Frederico Loureiro Bracarense, Regina Mitsuka-Breganó, Roberta Lemos Freire, Italmar Teodorico Navarro, Eloiza Teles Caldart

**Affiliations:** 1 Departamento de Medicina Veterinária Preventiva, Universidade Estadual de Londrina – UEL, Londrina, PR, Brasil

**Keywords:** Nematode, trichinellosis, artificial digestion, zoonosis, Nematódeos, trichinelose, digestão artificial, zoonose

## Abstract

*Trichinella* spp. are zoonotic parasites that are widely distributed in warm-blooded carnivores and omnivores, including humans. Until the present moment, Brazil has been considered by World Animal Health Organization free from the domestic cycle of trichinellosis, whereas the parasite’s sylvatic cycle has the status of infection in limited zones. However, neighboring countries such as Argentina have reports of parasite larvae in the wild fauna. The present study aimed to determine the occurrence of *Trichinella* spp. in road-killed wild animals in Paraná, Brazil. Biological samples from 71 wild animals—29 *Didelphis albiventris*, 11 *Nasua nasua*, ten *Cerdocyon thous*, seven *Dasypus novemcinctus*, six *Leopardus guttulus*, six *Sphiggurus spinosus* and two *Puma concolor*—collected from November 2016 to November 2021 were subjected to artificial digestion, following the methodology described in the REGULATION (EC) No. 2075/2005. No *Trichinella* spp. larvae were detected in the carcasses of the road-killed wild animals. However, considering the wide spectrum of possible reservoirs that could act as a link between the sylvatic and domestic cycles and considering the current Brazilian status of sylvatic trichinellosis in limited zones, frequent monitoring of wild fauna remains necessary.

## Resumo

*Trichinella* spp. é um parasito zoonótico de distribuição mundial detectado em carnívoros e onívoros de sangue quente e humanos. Até o momento, o Brasil é considerado pela Organização Mundial de Saúde Animal livre do ciclo doméstico da doença, enquanto o ciclo silvestre do parasito tem status de infecção em áreas limitadas. No entanto, em países vizinhos como a Argentina, larvas do parasito foram encontradas na fauna silvestre. O presente estudo teve como objetivo determinar a ocorrência de *Trichinella* spp. em animais silvestres atropelados no Paraná, Brasil. Amostras biológicas de 71 animais silvestres – 29 *Didelphis albiventris*, 11 *Nasua-nasua*, dez *Cerdocyon thous*, sete *Dasypus novemcinctus*, seis *Leopardus gattulus*, seis *Sphiggurus spinosus* e dois *Puma concolor*– coletadas de novembro de 2016 a novembro de 2021 foram submetidas à digestão artificial, seguindo a metodologia descrita do REGULAMENTO (CE) Nº2075/2005. Não foram encontradas larvas de *Trichinella* spp. nas carcaças dos animais selvagens atropelados. No entanto, considerando o amplo espectro de possíveis reservatórios que podem atuar como elo entre os ciclos silvestre e doméstico e considerando a condição atual da triquinelose silvestre no Brasil em áreas limitadas, o monitoramento frequente da fauna silvestre é necessário.

*Trichinella* spp. is a zoonotic intracellular nematode with global distribution that infects warm-blooded carnivores and omnivores, causing trichinellosis. *Trichinella spiralis* is the most prevalent species in human infections and is transmitted mainly by the consumption of raw or undercooked pork meat, salami, sausages, and bacon that contain larvae of the parasite. Furthermore, in many countries, infection can result from the ingestion of game animals (Gottstein et al., 2009; Rostami et al., 2017). Infection in humans occurs in two phases, intestinal and muscular, in which symptoms occur according to the infection stage, number of larvae ingested, and reproduction of adult nematodes in the small intestine mucosa. Symptoms are nausea, vomiting, transient diarrhea, myalgia, conjunctivitis, fever, headache, skin rash, and complications such as myocarditis, encephalitis, and thromboembolic disease (Gottstein et al., 2009).

Swine are infected by ingesting muscle tissue of animals with parasitic larvae, such as rodents and wild animals (Bruschi & Dupouy-Camet, 2014). In addition, although rare, equines can be accidentally infected through the consumption of pastures contaminated with carcasses of small rodents. As horse meat can be used an alternative source of protein, it may be a source of infection for humans in countries that consume this meat (Rostami et al., 2017). Trichinellosis is considered a serious public health issue that negatively impacts the pork market, as it is on the list of mandatory notifiable diseases of the World Organization for Animal Health (OIE). Therefore, when the occurrence of *Trichinella* spp. is confirmed, international trade of meat products is embargoed (Pozio, 2015).

The parasite is maintained in the environment through two main cycles of predation of carcasses containing parasite larvae: the domestic cycle, in which pigs and rodents are the main hosts of *T. spiralis*, and the sylvatic cycle, which involves warm-blooded carnivores and omnivores hosting several species of *Trichinella*, including *T. britovi, T. murrelli, T. nativa, T. nelson, T papuae, T. patagoniensis, T. pseudospiralis, T. spiralis e T. zimbabwensis* (Krivokapich et al., 2012). The disease’s status in wild animals in Brazil recently changed from “never reported” to “infection in limited zones” (WAHIS OIE, 2016). Considering the possibility of transmission and maintenance of the disease between the domestic and sylvatic cycles, the present study aimed to determine the occurrence of *Trichinella* spp. in road-killed wild animals in the state of Paraná, Brazil.

**Figure 1:**
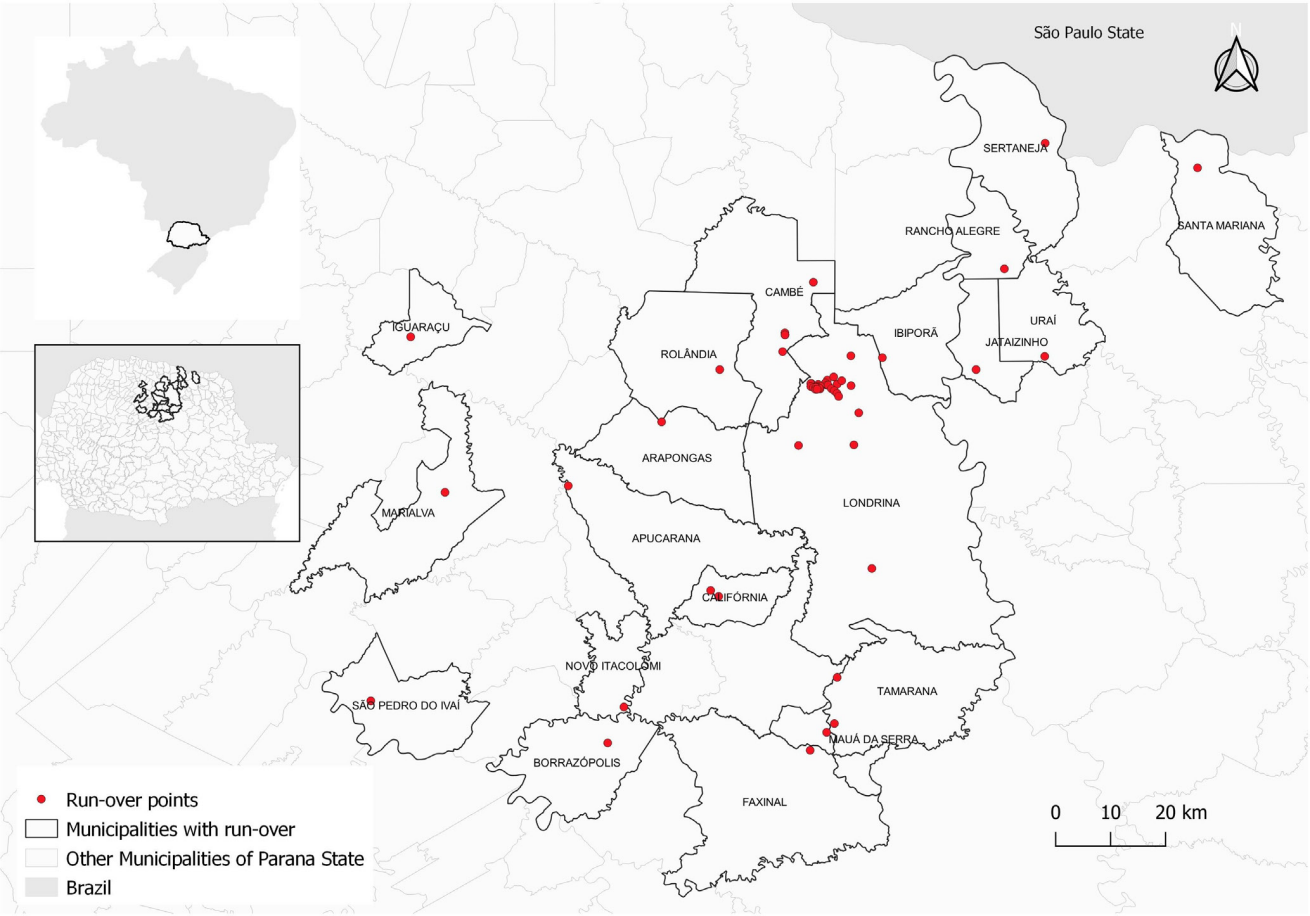
Biological samples from 71 road-killed wild animals collected between November/2016 and November/2021 from the northem region of the state of Paraná, Brazil.

The study was approved by the Biodiversity Authorization and Information System (SISBio) of the Environmental Institute (number 75977-1 and 55384-1) and by the Ethics Committee in the Use of Animals of the State University of Londrina (number 30/2017 and 130/2020). Biological samples from 71 road-killed wild animals collected between November 2016 and November 2021 from the northern region of the state of Paraná, Brazil, were obtained as described by Caldart et al. (2021). The collected animals were mapped using a Global Positioning System (GPS) and the point distributions were plotted using QGIS 2.14 software (Figure 1). Among the carnivores and omnivores, the carcasses of 29 *Didelphis albiventris*, 11 *Nasua nasua*, ten *Cerdocyon thous*, seven *Dasypus novemcinctus,* six *Leopardus guttulus,* six *Sphiggurus spinosus* and two *Puma concolor* were used. From each animal, an average of 5 g of diaphragm, masseter, and tongue fragments were packaged separately in freezer bags under freezing conditions. At the end of the collection period, a survey of *T. spiralis* larvae was performed using artificial digestion, following the methodology described in Regulation (EC) No. 2075/2005 (EU, 2005), which establishes specific official rules for the control of trichinellosis in meat.

The samples were pooled according to species and collection period, with a maximum of 10 animals processed at a time, and each set consisted of three muscle fragments (masseter, tongue, and diaphragm). The fragments were crushed and subjected to digestion with 25% hydrochloric acid and 1:10,000 pepsin under agitation at 46 °C for 30 min to release larvae from the musculature. The product was filtered to retain the undigested tissues and placed in a decantation flask for 30 min. A 40-mL sample was dispensed into a graduated cylinder for further sedimentation for 10 min. Finally, 30 mL of the supernatant were discarded, and the remaining 10 mL were used to count the larvae in a petri dish under a stereomicroscope Motic SMZ 168 model, using 1.0 to 7.5 magnification.

No *Trichinella* spp. larvae were detected in the carcasses of the road-killed wild animals in the present study.

According Caldart et al. (2021), the sampling of road-killed wild animals is essential for studies related to public health and conservation of native species. Research and monitoring of *Trichinella* spp. in wild fauna are necessary to deepen the knowledge about trichinellosis in the sylvatic cycle in Brazil, especially considering the wide spectrum of potential reservoirs that link the sylvatic and domestic cycles.

The detection of *Trichinella* spp. could be performed during the *post-mortem* inspection through artificial digestion of carcasses of species used for human consumption (Gottstein et al., 2009), this method may also be applied for detecting larvae in wildlife reservoirs (Nöckler et al., 2000). Artificial digestion method was selected as it allows pooling muscle samples of different individuals of the same species (Kapel et al., 2005). This technique is highly sensitive and efficient for detecting 1-3 larvae/g of digested muscle tissue (Gamble et al., 2000); however, sample size in studies on reservoir animals should be adjusted to >5 g, as their larval density is typically lower (Boireau et al., 2007).

European countries, such as Poland and Spain, have a higher prevalence of trichinellosis in humans, mainly associated with the consumption of wild boar meat from hunting (Moral et al., 2022). With the exception of Mexico, *Trichinella* spp. is widely distributed in North America, mainly observed in polar bears, red foxes, wild boars and puma (Crisóstomo-Jorquera & Landaeta-Aqueveque, 2022).

Trichinellosis has also been reported in South America in *Sus scrofa domesticus*, *Canis lupus familiaris*, *Felis catus*, *Chaetophractus villosus*, *Otaria flavescens*, *Puma concolor*, *Rattus norvegicus*, and *Lycalopex gymnocercus gracilis*, which inhabit wild and agricultural environments (Ribicich et al., 2021). Echeverry et al. (2021) found approximately 10 larvae/g of sample in the carcass of an illegally hunted *Puma concolor* in Chile using artificial digestion. The aforementioned direct test was used to detect *Trichinella* spp. in wild animals in Argentina, it was found in *Didelphis albiventris* and *Lutreolina crassicaudata* (Castaño Zubieta et al., 2014). *Didelphs albiventris* is a synanthropic animal widely distributed throughout the America, its epidemiological relevance refers to its ability to adapt to different environments, linking the environment of wild, rural and urban animals, including humans (Perez Carusi et al., 2009).

Our negative result is similar with other studies performed in Brazil, such as that by Catão et al. (1975), who also used the artificial digestion method to analyze 6,452 diaphragms of adult swine from the states of Paraná, Minas Gerais, São Paulo, and Goiás and found no larvae. Subsequently, Paim & Côrtes (1979) examined 594 diaphragms of *Rattus norvegicus* from the port area of Santos, São Paulo state, using the trichinoscopy technique and detected no parasite larvae. In the same way, Daguer et al. (2005) performed artificial digestion of different muscle samples of 3,774 adult pigs from 68 municipalities in the southern region of Paraná, all of them with negative results. Salazar & Salotti-Souza (2017) evaluated the presence of *T. spiralis* in 14,852 horses slaughtered in Araguari, Minas Gerais, Goiás, and Bahia using artificial digestion of muscle samples and found no larvae. Nonetheless it is important to highlight that herbivorous animals are considered uncommon hosts for this parasite.

Brazil is considered free from the domestic cycle of *Trichinella* spp. as the absence of the parasite has been reported in different animal species. Besides that, for the export of pork and equine meat to European Union countries, Brazil follows procedures defined in complementary standards (Decreto Nº 9.069 of May 31 – Brasil, 2017) that include *post-mortem* inspection of systematically sampled carcasses and sample collection for diagnosis following the European Union Regulation (EC) No. 2075/2005 (EU, 2005)

On the other hand, in 2016 the Brazilian status of ”never reported” for wild animals was replaced by “infection in limited zones”. The status has been changed after an official communication to governmental agencies of serological evidence of exposition to the parasite in wild boars, in vigilance from São Paulo, Mato Grosso, Santa Catarina and Rio Grande do Sul states by indirect ELISA (WAHIS OIE, 2016) Recently, 115 samples of wild boar sera from the Southeast region of Brazil were submitted to an indirect ELISA test validated for wild boars and seven animals were reactive (6.1%). None of the serologically positive animals were tested by enzymatic digestion due to the lack of sample. In the same study, carcasses of 37 wild boars and 15 carnivores (six canids and nine felids) were subjected to the enzymatic digestion test, all of them resulted negative for the parasite (Silva et al., 2022).

Summarizing, surveillance in domestic animals intended for human consumption has been carried out systematically in Brazil and presents robust results regarding the low level of infection for *Trichinella* in these species. On the other hand, serological evidence in wild boar draws attention to the need of intensify surveillance on these animals.

Furthermore, considering the wide spectrum of possible reservoirs that could act as a link between the sylvatic and domestic cycles and considering the current Brazilian status of sylvatic trichinellosis of infection in limited zones, frequent monitoring of wild fauna remains necessary.

## Ethics declaration

The study was approved by the Biodiversity Authorization and Information System (SISBio) of the Environmental Institute (number 75977-1 and 55384-1) and by the Ethics Committee in the Use of Animals of the State University of Londrina (number 30/2017 and 130/2020)

## Conflict of interest

The authors declare no conflicts of interest.
